# Knockdown of CMTM3 promotes metastasis of gastric cancer via the STAT3/Twist1/EMT signaling pathway

**DOI:** 10.18632/oncotarget.8789

**Published:** 2016-04-18

**Authors:** Wanqiong Yuan, Ting Li, Xiaoning Mo, Xiaolin Wang, Baocai Liu, Wenyan Wang, Yu Su, Lan Xu, Wenling Han

**Affiliations:** ^1^ Peking University Center for Human Disease Genomics, Department of Immunology, Key Laboratory of Medical Immunology, Ministry of Health, School of Basic Medical Sciences, Peking University Health Science Center, Beijing 100191, China

**Keywords:** CMTM3, gastric cancer, EMT, metastasis, STAT3

## Abstract

CMTM3 (CKLF-like MARVEL transmembrane domain containing 3) possesses tumor suppressor properties in multiple types of malignancies. Restoration of CMTM3 significantly inhibits the metastasis of gastric cancer, and its expression level is correlated with prognosis. However, the physiological effects and the mechanism of CMTM3 remain unknown. Here, we suppress CMTM3 expression by shRNA to explore its endogenous effects and its mechanism of action in gastric cancer. Stable knockdown of CMTM3 promotes cell migration, invasion and tumor metastasis, increases MMP2 expression and enhances MMP2 activity. CMTM3 inhibits EMT along with the upregulation of E-cadherin and the downregulation of N-cadherin, Vimentin and Twist1. It has no obvious effects on Zeb1 and Snail. CMTM3 suppresses the phosphorylation of STAT3 but not Akt. More importantly, the EMT phenotype and cell migration induced by CMTM3 knockdown can be reversed by the Jak2/STAT3 inhibitor JSI-124 or by siRNA against STAT3 or Twist1. Overall, this study demonstrates that knockdown of CMTM3 promotes the metastasis of gastric cancer through the STAT3/Twist1/EMT pathway.

## INTRODUCTION

CKLF-like MARVEL transmembrane domain-containing family (CMTM) is a novel family of proteins that links classical chemokines and the transmembrane-4 superfamily [1]. In humans, *CMTM* comprises nine genes, which are *Chemokine-like factor* (*CKLF*) and *CMTM1-8* [2]. CMTM family plays important roles in tumorigenesis. CMTM3, 5, 7 and 8 exhibit tumor suppressive properties in multiple types of malignancies, such as hepatocellular carcinoma [3], oral squamous carcinoma [4], esophageal squamous cell carcinoma [5], renal cell carcinoma [6], cervical cancer [7], testicular cancer [8], prostate cancer [9] and oral squamous cell carcinoma [10].

*CMTM3* locates on 16q22.1, an important tumor suppressor locus that is involved in multiple tumors. CMTM3 expression is reduced or silenced by methylation in gastric cancer cell lines and in primary gastric cancer tissues [11, 12]. Restoration of CMTM3 significantly suppresses the migration and invasion of gastric cancer cells, inhibits tumor metastasis *in vivo*, downregulates the expression of matrix metalloproteinase (MMP)2, reduces MMP2 activity and decreases the phosphorylation of Erk1/2. The prognosis of gastric cancer patients whose tumors express CMTM3 is better than those whose tumors lack CMTM3 expression [12]. However, the physiological effects and the mechanism of CMTM3 in the metastasis of gastric cancer remain unknown.

Gastric cancer ranks as the third leading cause of cancer mortality worldwide and is a leading cause of cancer death in less developed countries [13]. Metastasis is not only a sign of deterioration but is also a major cause of treatment failure in patients with gastric cancer [14]. EMT is a critical process in the metastatic cascade, and it has been established as a key regulator in many types of cancers including gastric cancer [15]. Recently, the mechanisms of EMT were further elucidated. EMT transcription factors Zeb1 [16, 17], Snail [17, 18] and Twist1 [18, 20] are important players in the regulation of EMT during cancer metastasis through different signaling cascades, including the Akt [22, 22], Erk1/2 [22] and signal transducer and activator of transcription 3 (STAT3) [23] signaling pathways. During EMT, the epithelial-specific junction protein, E-cadherin is downregulated and mesenchymal proteins such as N-cadherin, Vimentin and MMP-2 are upregulated [17, 24]. The metastasis of gastric cancer may be restrained by the inhibition of EMT. However, whether these signaling pathways influence the metastasis of gastric cancer is largely unknown.

In this paper, we investigated the effects and the mechanism of endogenous CMTM3 in human gastric cancer cells using lentiviral short hairpin RNA (shRNA). We found that knockdown of CMTM3 promoted cell migration, invasion and tumor metastasis via the STAT3/Twist1/EMT pathway, which will be helpful to understand the pathogenesis of gastric cancer.

## RESULTS

### Knockdown of CMTM3 promotes cell migration and invasion, upregulates MMP2 expression and enhances MMP2 activity

**Figure 1:**
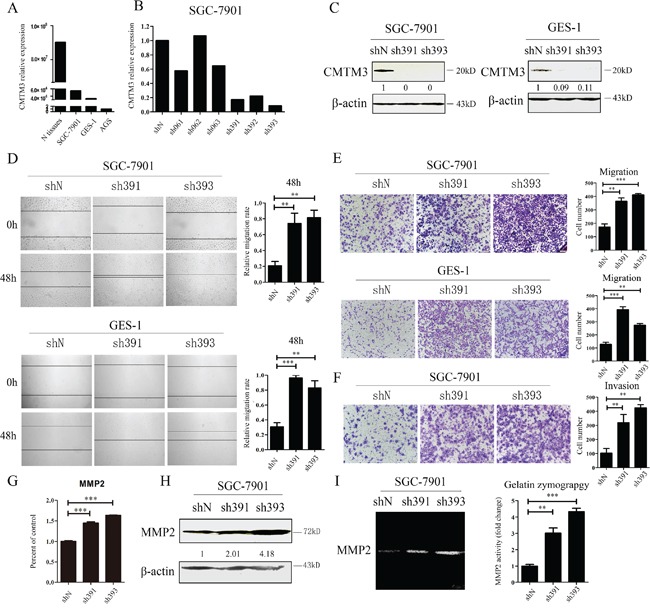
Knockdown of CMTM3 promotes cell migration and invasion **A.** expression of CMTM3 in the gastric mucosa (N tissues), SGC-7901, GES-1 and AGS cells was detected by real-time PCR. **B.** SGC-7901 cells were infected with six CMTM3 shRNAs, which are named according to the last three cloning item numbers: sh061, 062, 063, 391, 392, 393. The expression of CMTM3 was analyzed by real-time PCR. **C.** SGC-7901 (left) and GES-1 (right) cells were infected with the lentivirus supernatants: shN, sh391 or sh393, and CMTM3 expression was analyzed by western blot. The average relative gray density is shown under each blot. **D.** wound-healing assay in shN, sh391 and sh393 of SGC-7901 (upper) and GES-1 (lower) cells; photographs were obtained at the indicated time points after the scratch was generated (100 × magnification). **E.** Transwell assay of shN, sh391 and sh393 of SGC-7901 cells (upper) and GES-1 cells (lower); the cells were fixed and stained with crystal violet after 12 h (SGC-7901) or 24 h (GES-1) of incubation and photographs were obtained (100 × magnification). **F.** Transwell assay to assess the invasion of shN, sh391 and sh393 of SGC-7901 cells; cell numbers were counted and photographs were obtained after 48 h of incubation (100 × magnification). **G.** the expression of MMP2 was detected by real-time PCR in shN, sh391 and sh393 of SGC-7901 cells. **H.** the expression of MMP2 was detected by western blot in shN, sh391 and sh393 of SGC-7901 cells. The average relative gray density is shown under each blot. **I.** MMP2 activity was observed by Gelatin Zymography assay in shN, sh391 and sh393 of SGC-7901 cells. Data are presented as the mean±s.d (**P<0.01, ***P<0.001).

Earlier studies have indicated that CMTM3 is downregulated or silenced in gastric cancer cell lines and in primary tumor tissues [12]. To study the physiologic functions of CMTM3, we analyzed and compared its expression in GES-1 cells, an immortalized gastric epithelial cell line with AGS cells, SGC-7901 cells and normal gastric mucosal tissues at the mRNA level by real-time PCR. As shown in Figure 1A, the expression level of CMTM3 in GES-1 cells was slightly lower than that in SGC-7901 cells, but was much higher than that in AGS cells. Thus, the SGC-7901 and GES-1 cell lines were chosen to study the endogenous role of CMTM3. To specifically and stably knock down the expression of CMTM3, six different nucleotide sequences were designed for shRNAs. The knockdown efficiency of CMTM3 was detected by real-time PCR in SGC-7901 cells (Figure 1B). Almost all shRNAs blocked CMTM3 expression compared with shN. Notably, the knockdown efficiency of sh391 and sh393 was better than the others (typically more than 80%). We further confirmed the knockdown efficiency at the protein level in SGC-7901 cells (Figure 1C, left) and GES-1 cells (Figure 1C, right).

Previous data have shown that restoration of CMTM3 significantly attenuates migration and the invasiveness of gastric cancer cells [12]. To examine the effects of endogenous CMTM3 on cell migration, a wound-healing assay was performed in both SGC-7901 (Figure 1D, upper) and GES-1 (Figure 1D, lower) cells after knockdown of CMTM3. We observed that the wound edge of the CMTM3-knockdown cells was markedly closer than that of the shN cells. The results of Transwell assays revealed a significant increase in migration of sh391 and sh393 cells compared with shN cells in both SGC-7901 (Figure 1E, upper) and GES-1 cells (Figure 1E, lower). To further explore the role of CMTM3 in cell invasion, a Transwell assay was performed in SGC-7901 cells with Matrigel pre-coated in the chamber. As shown in Figure 1F, CMTM3-knockdown cells displayed a markedly stronger invasive capacity compared with the control cells. Overall, these results suggested that knockdown of CMTM3 promotes cell migration and invasion *in vitro*.

MMPs, especially MMP2, can degrade the basement membrane and extracellular matrix to regulate cell migration and invasion. Earlier studies have shown that restoration of CMTM3 inhibits MMP2 expression and suppresses its activities [12]. To further confirm the mechanism of the accelerated cell invasion that was observed after knockdown of CMTM3, MMP2 expression was detected in SGC-7901 cells. It was observed that sh391 and sh393 cells expressed more MMP2 than shN cells (Figure 1G, 1H). A Gelatin Zymography assay further indicated that MMP2 activity was significantly enhanced by silencing CMTM3 (Figure 1).

We also examined cell proliferation after knockdown of CMTM3. As illustrated in Supplementary Figure 1, CMTM3 exerts no obvious impact on the proliferation of gastric cancer cells both in anchorage-dependent and independent conditions.

### Knockdown of CMTM3 promotes peritoneal metastasis of gastric cancer *in vivo*

**Figure 2:**
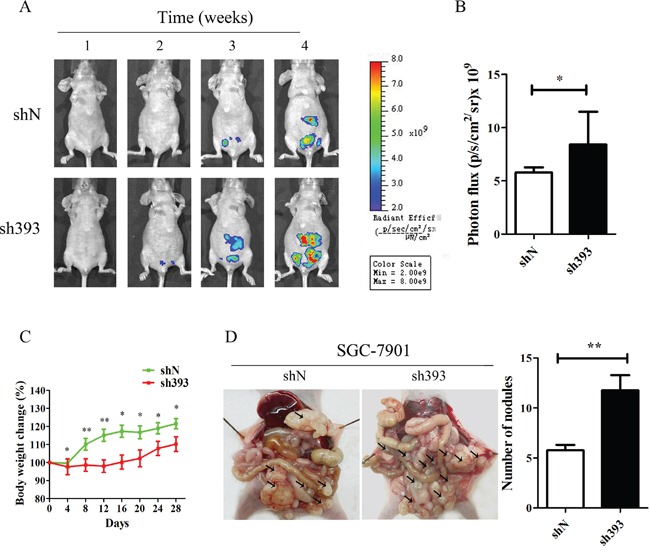
Silencing CMTM3 promotes tumor metastasis *in vivo* A total of 4×10^6^ cells (SGC-7901-shN or SGC-7901-sh393, n=8 per group) was injected into the abdominal cavity of each mouse on Day 0. **A.** fluorescence visualization of tumors from representative mice from each group was detected by an IVIS Spectrum Imaging System. **B.** fluorescence intensity was quantified and shown by the bar graphs. **C.** the changes in body weight were monitored every four days after injection. **D.** images of the peritoneal cavity were obtained after the mice were sacrificed. Arrows indicate the disseminated tumor nodules. Disseminated nodules larger than or equal to 1 mm in diameter were counted and were shown in the bar graphs. Data are presented as the mean±s.d (*P<0.05 **P<0.01).

*In vitro* experiments have demonstrated that silencing CMTM3 promoted cell migration and invasion. To further confirm the effects of CMTM3 on tumor metastasis, an animal model of peritoneal metastasis was established by the intraperitoneal injection of SGC-7901-shN or SGC-7901-sh393 cells into mice. As shown in Figure 2A, a fluorescence signal could be detected after two weeks in the sh393 group, while no fluorescence signal was detected in the control shN group. Although fluorescence was eventually detected in the shN mice, the fluorescence intensity was far weaker than that in the sh393 mice. The quantified fluorescence intensity was represented by bar graphs as shown in Figure 2B. The body weight of the mice was monitored every four days from the time of injection. The changes revealed that knockdown of CMTM3 promoted metastasis, which further caused a decrease in the body weight of the mice (Figure 2C). All mice were sacrificed 4 weeks after injection. As shown in Figure 2D, a higher number of metastatic nodules were found in the peritoneal cavity of the sh393 group compared with the shN group. Combined with the *in vitro* findings, we conclude that the knockdown of CMTM3 promotes cell migration, invasion and metastasis of gastric cancer cells.

### Twist1 is involved in CMTM3-suppressed EMT in gastric cancer

**Figure 3:**
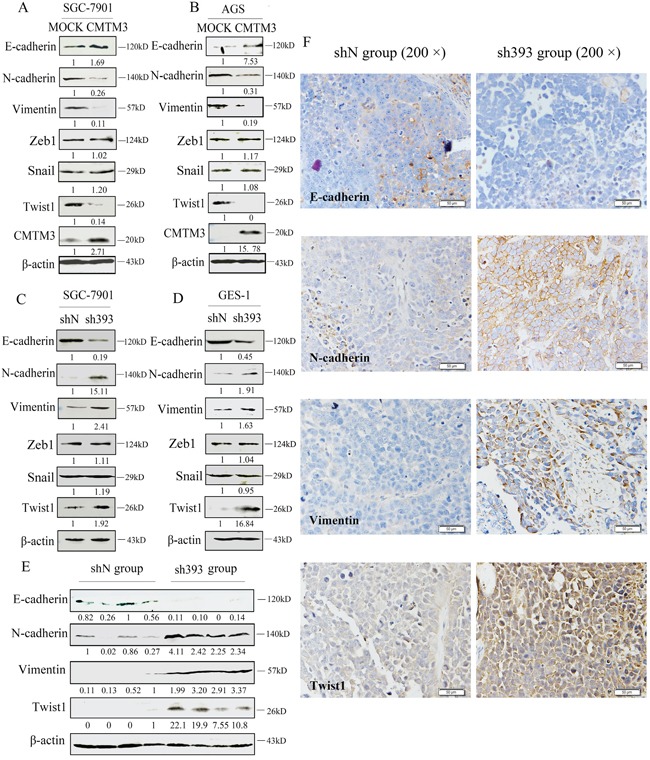
Twist1 plays a vital role in the inhibition of EMT by CMTM3 **A.** expression of the EMT markers E-cadherin, N-cadherin, Vimentin, Zeb1, Snail and Twist1 was analyzed in CMTM3-restored SGC-7901 cells (MOI: 100). **B.** expression of EMT markers was analyzed in CMTM3-restored AGS cells (MOI: 100). **C.** expression of EMT markers was examined in shN and sh393 of SGC-7901 cells. **D.** expression of EMT markers was examined in shN and sh393 of GES-1 cells. **E.** expression of EMT markers was analyzed in tumors from mouse model of peritoneal metastasis (4 mice were randomly selected from the 8 mice in each group) by western blot. **F.** representative IHC staining for the EMT markers E-cadherin, N-cadherin, Vimentin, and Twist1 (top, shN group; bottom, sh393 group, 200 × magnification) was performed. The average relative gray density is shown under each blot (A-E).

Overexpression of CMTM3 in SGC-7901 and AGS cells inhibits cell migration and invasion [12], but the mechanism remains to be studied. In the current study, we further demonstrated that silencing CMTM3 promoted cell migration and invasion. Next, we aimed to investigate the effects of CMTM3 on EMT, which is important in the initiation and promotion of cell migratory and invasive properties [14]. First, the expression of EMT markers was analyzed in CMTM3 restored SGC-7901 cells and AGS cells. As shown in Figure 3A and 3B, overexpression of CMTM3 increased the expression of E-cadherin but decreased the expression of N-cadherin and Vimentin. Among the EMT-related transcription factors, the expression of Zeb1 and Snail was not obviously affected. However, Twist1 expression was significantly downregulated. Further, we confirmed the effects of CMTM3 on EMT in CMTM3-knockdown SGC-7901 cells (Figure 3C) and GES-1 cells (Figure 3D). The expression of E-cadherin was downregulated whereas the expression of N-cadherin, Vimentin and Twist1 was upregulated, but no obvious differences were observed in the expression of Zeb1 and Snail.

Then, the expression of EMT markers was analyzed in tumor samples from *in vivo* peritoneal metastases model by western blot (Figure 3E) and IHC staining (Figure 3F). The expression of E-cadherin was clearly downregulated, while the expression of N-cadherin, Vimentin and Twist1 was upregulated. Together, these changes suggested that Twist1 is a crucial transcription factor in the inhibition of EMT by CMTM3.

### Knockdown of CMTM3 promotes cell migration in a STAT3-dependent manner

**Figure 4:**
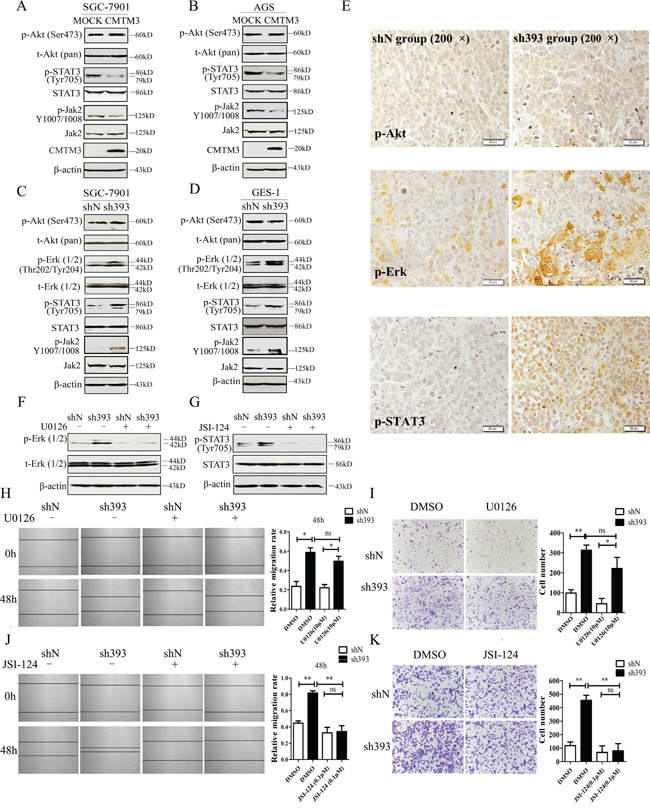
The STAT3 signaling pathway plays a crucial role in promoting migration of CMTM3-knockdown SGC-7901 cells **A-B.** the phosphorylated and total Akt, STAT3 and Jak2 were analyzed in CMTM3 restored cells by western blot. **C-D.** the phosphorylated and total Akt, Erk1/2, STAT3 and Jak2 were analyzed in CMTM3 knockdown cells by western blot. **E.** representative IHC staining for p-Akt, p-Erk1/2 and p-STAT3 in tumors from mouse model of peritoneal metastasis (top, shN group; bottom, sh393 group, 200 × magnification) was performed. **F.** shN- and sh393-SGC-7901 cells were treated with the Erk1/2 inhibitor U0126 (10 μM) for 12 hours, and the level of p-Erk1/2 was analyzed by western blot. **G.** shN- and sh393-SGC-7901 cells were treated with the Jak2/STAT3 inhibitor JSI-124 (0.1 μM) for 24 hours, and the level of p-STAT3 was analyzed by western blot. **H.** wound-healing assay in shN and sh393 of SGC-7901 cells in the presence or absence of U0126 (10 μM); photographs were obtained at the indicated time points after the scratch was generated (100 × magnification). **I.** A Transwell assay of SGC-7901 cells in the presence or absence of U0126 (10 μM); photographs were obtained after 12 hours of incubation (100 × magnification). **J.** wound-healing assay in shN and sh393 of SGC-7901 cells in the presence or absence of JSI-124 (0.1 μM); photographs were obtained at the indicated time points after the scratch was generated (100 × magnification). **K.** A Transwell assay of SGC-7901 cells in the presence or absence of JSI-124 (0.1 μM); photographs were obtained after 12 hours of incubation (100 × magnification). Data are presented as the mean±s.d (*P<0.05 **P<0.01).

To elucidate the mechanisms that underlie the inhibition of tumor metastasis and EMT by CMTM3, the Akt, Erk1/2 and STAT3 signaling pathways, which play important roles in cell migration, tumor metastasis and EMT, were analyzed. The levels of total and phosphorylated Akt (Ser473) and STAT3 (Tyr705) were detected in CMTM3 overexpressed SGC-7901 (Figure 4A) and AGS cells (Figure 4B), and phosphorylated Akt (Ser473), Erk1/2 (Thr202/Tyr204) and STAT3 (Tyr705) were detected in knockdown SGC-7901 (Figure 4C) and GES-1 cells (Figure 4D). No significant difference was observed in the level of p-Akt in these cells, but p-Erk1/2 was increased in CMTM3 knockdown cells, which was in accordance with our overexpression results [12]. Moreover, p-STAT3 was significantly decreased in CMTM3 restored SGC-7901 and AGS cells, and increased in CMTM3 knockdown SGC-7901 and GES-1 cells. It has been well studied that p- Janus-activated kinase 2 (Jak2) could activate STAT3, and the Jak2/STAT3 signaling pathway is involved in the development of cancers and EMT [25]. Thus, we detected the effect of CMTM3 on p-Jak2 and found that p-Jak2 was obviously decreased in CMTM3 restored cells and increased in CMTM3 knockdown cells (Figure 4A–4D). Further, we confirmed the status of p-Akt, p-Erk1/2 and p-STAT3 in tumors from the peritoneal metastases mouse model by IHC. Similarly, no significant differences in p-Akt were found between the two groups, while higher levels of p-Erk1/2 and p-STAT3 were observed in sh393 mice (Figure 4E). To explore the roles of the Erk1/2 and STAT3 signaling pathways in cell migration after knockdown of CMTM3, the Erk1/2 inhibitor U0126 (10 μM) or the Jak2/STAT3 inhibitor JSI-124 (0.1 μM) was applied. The levels of p-Erk1/2 (Thr202/Tyr204) (Figure 4F) and p-STAT3 (Tyr705) (Figure 4G) were dramatically reduced by their respective inhibitors. According to the wound healing assay, treatment with the Erk1/2 inhibitor U0126 did not abrogate the tumor-promoting properties induced by knockdown of CMTM3 (Figure 4H). Transwell assay revealed that although U0126 could attenuate cell migration in both shN and sh393 cells, it did not reverse the cell migration promoted by silence of CMTM3 (Figure 4I). However, treatment with the Jak2/STAT3 inhibitor JSI-124 completely inhibited the migration of SGC-7901 cells promoted by knockdown of CMTM3 in both the wound healing assay (Figure 4J) and the Transwell assay (Figure 4K). Thus, we conclude that silencing CMTM3 promotes cell migration in a STAT3-dependent manner.

### Knockdown of CMTM3 induces gastric cancer cell migration via the STAT3/Twist1/EMT pathway

**Figure 5:**
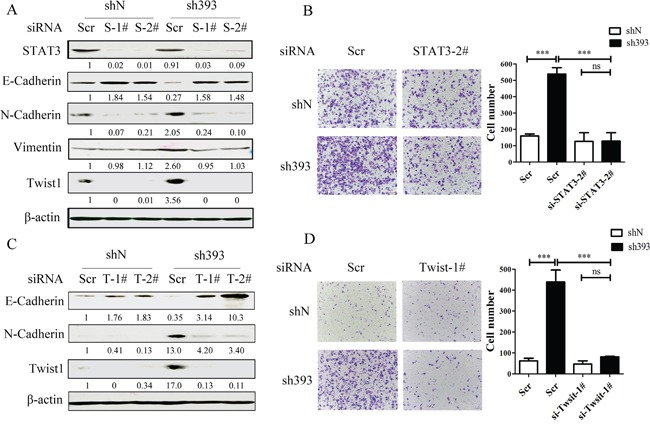
CMTM3-knockdown-induced cell migration is dependent on the STAT3/Twist1/EMT pathway **A.** SGC-7901-shN and SGC-7901-sh393 cells were transfected with control siRNA (Scr), si-STAT3-1^#^ or 2^#^ (S-1^#^ or S-2^#^). The expression of STAT3 and EMT markers was analyzed by western blot. **B.** A Transwell assay was performed in SGC-7901-shN and SGC-7901-sh393 cells that were transfected with control siRNA (Scr) or with si-STAT3-2^#^. Photographs were obtained after 24 hours of incubation (100 × magnification). **C.** SGC-7901-shN and SGC-7901-sh393 cells were transfected with control siRNA (Scr) or with si-Twist1-1^#^ or 2^#^. (T-1^#^ or T-2^#^). The Twist1 and EMT markers were analyzed by western blot. **D.** A Transwell assay was performed in SGC-7901-shN and SGC-7901-sh393 cells that were transfected with Scr or with si-Twist-1^#^. Photographs were obtained after 24 hours of incubation (100 × magnification). The average relative gray density is shown under each blot (A, C). Data are presented as the mean±s.d (***P<0.001).

It has been reported that STAT3 plays important roles in the regulation of cell migration as well as in the initiation and resolution of EMT [18, 26]. The above data indicated that the Jak2/STAT3 inhibitor could abolish the cell migration induced by CMTM3 knockdown. To clarify the role of STAT3, siRNAs were utilized in CMTM3-knockdown cells. The transfection efficiency of the positive control siFAM was approximately 98.6% as measured by flow cytometry. We silenced STAT3 by siRNA and assessed the expression of the transcription factor Twist1 and other three EMT markers in SGC-7901-sh393 and SGC-7901-shN cells. As exhibited in Figure 5A, knockdown of STAT3 led to the upregulation of E-cadherin expression and the downregulation of N-cadherin, Vimentin and Twist1 expression. Silence of STAT3 also clearly attenuated the migration of sh393-si-STAT-2^#^ cells compared with sh393-Scr cells (3.4-fold) (Figure 5B). These results further confirmed that the cell migration-promoting effects after the knockdown of CMTM3 are dependent on the STAT3 signaling pathway.

Figure 3 showed that among the EMT transcription factors Zeb1, Snail and Twist1, only Twist1 expression was affected by CMTM3. Twist1 is one of the core EMT-promoting proteins and is a downstream target of the STAT3 signaling pathway, which is responsible for metastasis of different tumors [19, 20, 27, 28]. Moreover, Cheng GZ et al. found that STAT3 could bind to the Twist1 promoter and activate its transcription [29]. To explore the roles of Twist1 in CMTM3-knockdown-induced metastasis and EMT, Twist siRNAs were used. Cells treated with Twist1 siRNAs showed higher levels of E-cadherin and lower levels of N-cadherin compared with control Scr cells (Figure 5C). Silence of Twist1 also reduced the migratory ability promoted by knockdown of CMTM3 in these cells. The number of migrated sh393-si-Twist1-1^#^ cells was obviously less than that of the sh393-Scr cells (approximately 5.5-fold) (Figure 5D). These changes revealed that silence of Twist1 could reverse CMTM3-knockdown-induced EMT. Taken together, knockdown of CMTM3 promoted cell migration though the STAT3/Twist1/EMT pathway in gastric cancer.

## DISCUSSION

Our previous study showed that CMTM3 is silenced or downregulated in gastric cancer cell lines and in primary gastric cancer tissues. Restoration of CMTM3 inhibits the migration and metastasis of gastric cancer cells *in vitro* and *in vivo* [12]. Through a shRNA approach, we now focus on the tumor-suppressive contribution of endogenous CMTM3 and its mechanism. We found that knockdown of CMTM3 promotes metastasis and induces EMT via the STAT3/Twist1/EMT pathway.

In addition to STAT3, we also determined the role of CMTM3 in the Akt signaling pathway and verified the changes of Erk1/2 in the CMTM3 knockdown system. Knockdown of CMTM3 has no obvious effects on the phosphorylation of Akt, but causes a slight increase in the level of p-Erk1/2. Then, we analyzed the roles of the Erk1/2 signaling pathways in cell migration. The results showed that silence of Erk1/2 could not reverse the cell migration and EMT induced by knockdown of CMTM3. Considering that the Erk1/2 pathway plays critical roles in numerous cellular processes, including cell differentiation, proliferation and apoptosis in gastric cancer [30–32], we speculate that activated p-Erk1/2 in CMTM3-knockdown cells may influence other aspects of tumor progression in gastric cancer cells. Elevated expression of the EMT-inducing transcription factors Zeb1, Snail and Twist1 can promote EMT in multiple cancer types. Activated STAT3 can directly bind to the promoter of Snail [33] or Twist1 [29] to induce EMT in cancer cells. Moreover, Snail and Twist1 are overexpressed in gastric carcinoma [34]. Our results showed that out of these proteins, only Twist1 is involved in the CMTM3-suppressed EMT phenotype in gastric cancer cells. In summary, our findings demonstrated that CMTM3-knockdown-induced EMT is dependent on the activation of STAT3 and on the expression of Twist1.

EMT is a dynamic process that is triggered by cytokines, such as transforming growth factor-β and interleukin 6 (IL-6) [35, 36]. IL-6 is an activator of Jak2/STAT3 signaling pathway which is involved in the development of cancers and EMT [37, 38]. We found that CMTM3 significantly reduced p-Jak2 and p-STAT3. Thus, we further assessed the IL-6-induced Jak2 and STAT3 activation in CMTM3 restored and knockdown SGC-7901 cells. As illustrated in Supplementary Figure 2A, CMTM3 restored cells showed a reduced activation of Jak2/STAT3 after stimulation with IL-6. While CMTM3 knockdown cells promoted the activation of Jak2/STAT3 after IL-6 treatment (Figure 2B). Together, these observations indicate that CMTM3 regulates IL-6 induced Jak2 and STAT3 phosphorylation.

Besides IL-6, epidermal growth factor receptor (EGFR) could also activate the Jak2/STAT3 signaling pathway [39, 40]. Moreover, there is accumulating evidences that EMT may be triggered by growth factors such as epidermal growth factor, hepatocyte growth factor and platelet-derived growth factor [41–43]. Receptors of growth factors such as tyrosine kinase receptors (RTKs) play critical roles in the progression of gastric cancer [45–46]. Higher expression levels of RTKs were detected in gastric cancer tissues compared with normal tissues, and some RTKs (e.g., EGFR, HER2, c-MET and vascular endothelial growth factor receptor) have been known to induce invasion, metastasis and EMT in gastric cancer [47, 48]. It has been reported that some CMTM family members, such as CMTM7 [5] and CMTM8 [3] exert their roles in human cancers by influencing RTK and RTK-related signaling pathways. It is therefore necessary to further determine other upstream activators of the STAT3/Twist1/EMT pathway in the CMTM3-knockdown system and to investigate the function of CMTM3 on the expression, stabilization, internalization and degradation of RTKs in gastric cancer cells.

CMTM proteins have a conserved MARVEL (MAL Related proteins for Vesicle trafficking and membrane Link) domain [11]. Besides the CMTM family, MARVEL-related proteins include multiple protein families, such as Myelin and Lymphocyte, Physin, Claudin and Occludin [49]. Recent evidence supports that MARVEL-related proteins are closely associated with the progression of EMT. Claudin-1 and −7, tricellulin and marvelD3 are involved in EMT in pancreatic cancer cells [50]. Knockdown of CMTM8, another CMTM family member, induces EMT progression in hepatocyte carcinoma cells [3]. In this study, the inhibitory effects of CMTM3 on EMT in gastric cancer may extend our understanding of the function of the CMTM family and MARVEL-related proteins on EMT during carcinogenesis.

It has been reported that CMTM3 plays crucial roles as a tumor suppressor in other human cancers. In prostate cancer cells, CMTM3 expression is decreased by IL-30 (134-fold), which has been shown to have pro-tumor activities [9]. CMTM3 suppresses the proliferation, and migratory capacity of testicular cancer cells [8]. CMTM3 is also downregulated in 84% of renal cell carcinoma (63/75); Restoration of CMTM3 in a renal cell carcinoma cell line inhibits cell proliferation and migration [6]; CMTM3 expression is significantly reduced in OSCC cell lines and primary tumor specimens. CMTM3 inhibits cell growth and migration and predicts favorable survival in oral squamous cell carcinoma [10]. However, Sabit Delic et al. has proposed an opposite function for CMTM3. They found that CMTM3 is upregulated in glioblastoma tissues compared with non-neoplastic brain tissues and that knockdown of CMTM3 by siRNA reduces cell invasion [51]. According to the present studies, it seems that the expression profile and function of CMTM3 are tissue-specific. Moreover, it has been reported that mutations in tumors suppressor genes (TSGs) such as *p16* and *p53* may result in the loss of tumor-suppressor functions and the acquisition of oncogenic characteristics in multiple cancer types [52–55]. Therefore, it is necessary to verify whether CMTM3 is mutated in glioblastoma.

Our previous results showed that CMTM3 expression is strongly associated with gender, tumor depth, stage, and histological grade in 350 gastric cancer samples. The prognosis of patients with gastric cancer is better in those whose tumors express CMTM3 [12]. Based on the findings of the current study, it is necessary to analyze the relationship among CMTM3, p-STAT3, Twist1 expression and metastasis in gastric cancer. In summary, our data demonstrate that CMTM3 suppresses gastric cancer metastasis via the STAT3/Twist1/EMT pathway, which will be helpful in our understanding of the pathogenesis of gastric cancer and provide novel clues for the early diagnosis of metastasis.

## MATERIALS AND METHODS

### Cell lines and reagents

The human gastric cancer cell line SGC-7901 was obtained from the Cell Research Institute (Shanghai, China), AGS cells were obtained from ATCC (Manassas, VA, USA), and GES-1 was kindly provided by the Beijing Cancer Hospital. All of these cell lines were cultured in RPMI-1640 medium (Invitrogen, Carlsbad, CA, USA) supplemented with 10% characterized fetal bovine serum (FBS) (HyClone, Logan, UT, USA), 10 units/mL penicillin and 10 mg/mL streptomycin. HEK293T cells were kindly provided by T. Matsuda (Japan) and were cultured in DMEM with 10% FBS, 10 units/mL penicillin and 10 mg/mL streptomycin. All cells were maintained in humidified incubators at 37°C and 5% CO_2_. The Erk1/2 inhibitor U0126 (CST, MA, USA) and the Jak2/STAT3 inhibitor JSI-124 (Cayman, USA) were purchased commercially.

### Western blot and antibodies

Western blotting was performed as previously described [11]. Antibodies to MMP2, Erk1/2 and phosphor-Erk1/2 (Thr202/Tyr204), Akt (pan) and phosphor-Akt (Ser473), STAT3 and phosphor-STAT3 (Tyr705), Jak2 and phosphor-Jak2 (Y1007/1008), N-cadherin, Vimentin, Snail (CST, MA, USA), E-cadherin (BD, USA), Twist1 (SAB, USA), Zeb1 (Santa Cruz Biotechnology) and Ki67 (Abcam, Cambridge, UK) were purchased commercially. The rabbit anti-CMTM3 antibody was prepared and purified in our laboratory [12].

### Real-time PCR

Real-time PCR was performed as previously described [12].

### Adenovirus construction and cell infection

The construction, generation, purification and infection of the ad5-null (vector-containing adenovirus, defined as Mock) and ad5-CMTM3 vectors were performed as previously described [12].

### Lentivirus transduction

The pGIPZ-lentiviral shRNAmir vectors that target human CMTM3 and the nonsilencing pGIPZ control vector (shN) were synthesized by Open Biosystems (Thermo Fisher Scientific, Inc.). They each contain a Turbo green fluorescent protein (GFP) reporter and express a puromycin-resistant gene. The lentiviruses were packaged via the co-transduction of HEK293T cells with the lentiviral vector plasmid containing shRNAmir and the packaging plasmids psPAX2 and pLP/VSVG using Vigofect transfection reagent (Vigorous Biotechnology, China). Sixty hours after transfection, the supernatants that contained the lentivirus were collected. The target cells SGC-7901 and GES-1 were infected with the collected supernatants according to the manufacturer's protocol. A total of 400 ng/mL or 600 ng/mL of puromycin (Cellgro, USA) was added to the medium of SGC-7901 and GES-1 cells respectively to select the transfected cells. For a higher knockdown efficiency, the highest GFP-expressing cells were selected and obtained by cell sorting.

### siRNA transfection

To knock down endogenous STAT3 and Twist1, the following target sequences were constructed in a small interfering RNA (siRNA) vector: si-STAT3-1^#^, 5ʹ-GCAAGAUUCAGACCCUCAATT-3ʹ, si-STAT3-2^#^, 5ʹ-CCAACAAUCCCAAGAAUGUTT-3ʹ; si-Twist1-1^#^: 5ʹ-GCAAGAUUCAGACCCUCAATT-3ʹ, si-Twist1-2^#^: 5ʹ-GGAGUCCGCAGUCUUACGATT-3ʹ. A scrambled siRNA (Scr) sequence was used as a negative control: 5ʹ-UUCUCCGAACGUGUCACGUTT-3ʹ. All these siRNAs and the positive control siRNA FAM were purchased commercially (Gene Pharma Inc., Shanghai, China). Cells were transfected with siRNA 24 hours later at a final concentration of 50 nM by Lipofectamine 3000 transfection reagent (Life Technologies, Carlsbad, CA) for further experiments. The suppression efficiency of si-STAT3 or si-Twist1 was analyzed by western blot at 48 or 72 hours, respectively.

### Cell proliferation assay

Cell proliferation was measured by a Cell Counting Kit-8 (CCK8) detection kit (Dojindo Molecular Technologies, Japan). The cells were seeded at a concentration of 2000 cells per well in a 96-well plate. At the indicated time points, 10 μL CCK-8 solution was added to each well followed by an incubation at 37°C for 100 min. Absorbance values of all wells were then determined at 450 nm.

### Plate colony formation assay

The cells were seeded into a 6-well plate at a density of 400 cells per well with 400 ng/mL puromycin. The selective medium was replaced every 4 days. After 10 days, the cell colonies were fixed in 4% paraformaldehyde/PBS and stained with 2% crystal violet. The colonies (≥50 cells per colony) were counted and imaged under a light microscope.

### Soft-agar colony formation assay

The cells were seeded into a 12-well plate at a density of 500 cells per well and were suspended in RPMI-1640 with 0.3% agar and 10% FBS. The cells were layered in RPMI-1640 with 0.6% agar, 10% FBS and 400 ng/mL puromycin. After 2 weeks, the colonies were counted and imaged under a light microscope.

### Wound-healing assay

Cells were seeded in a 6-well plate and grown to 80% confluence. A wound was scrapped with a sterilized pipette tip in the middle of the cell monolayer carefully. Photomicrographs were taken 48 hours after scrapping. The wound width in the microscopic pictures were measured by ImageJ software version 1.37 (National Institutes of Health, USA) at different time points. The percentage of wound healing was calculated from the initial wound width at 0 h.

### Cell migration and invasion assay

Cell migration and invasion were analyzed in Transwell chambers (8-μm pore size, BD Biosciences, NJ, USA). The cells were cultured in serum-free RPMI-1640 medium overnight before the initiation of the experiments. The following day, 1×10^5^ (for the invasion assay) or 5×10^4^ (for the migration assay) cells in 0.25 mL serum-free RPMI-1640 medium were seeded into the upper chamber, which was pre-coated with (for the invasion assay) or without (for the migration assay) Matrigel. 0.5 mL RPMI-1640 with 10% FBS was added to the lower chamber. After incubation at 37°C, the chambers were disassembled, and the membranes were fixed in 4% paraformaldehyde/PBS for 10 min and stained with 2% crystal violet for 10 min. The number of cells was counted and images were obtained under a microscope (100 × magnification).

### Gelatin zymography

Cells were incubated for 16 hours in serum-free medium. The supernatants were collected for Gelatin Zymography. 0.1% gelatin was used as a substrate. Following electrophoresis, the gels were washed with the renaturing buffer (2.5% Triton X-100) and then incubated for 24 hours at 37°C in the developing buffer (10 mM CaCl2, 15 mM NaCl, 2 mM NaN3 and 50 mM Tris-HCl, pH 7.5). The gels were stained with Coomassie Blue for 1 hour and destained at an appropriate time. Odyssey Infrared Imager (LI-COR Bioscience) was used to scan the gels.

### Animal model of peritoneal metastasis

Five to six-week-old female BALB/c nude mice were purchased from Vital River (Beijing, China). The mice were bred and maintained under specific pathogen-free conditions, provided with sterilized food and water and housed in a barrier facility with a 12 h light/dark cycle. The mice were randomly divided into two groups and were injected with SGC-7901-shN (n=8) or -sh393 (n=8) cells that expressed TurboGFP. In all, 4×10^6^ cells were injected into the abdominal cavity of the mice along with 250μl PBS. The body weight of the mice was measured every 4 days. Tumor growth and metastasis were monitored *in vivo* via weekly measurement of the fluorescence intensity by the IVIS Spectrum Imaging System (PerkinElmer, USA). After all mice were sacrificed on day 28, the visible disseminated nodules were counted. The tumors were excised, cut into blocks, fixed in 10% formalin, and embedded in paraffin for IHC or were snap-frozen in liquid nitrogen for western blot.

### Immunohistochemistry

Immunohistochemistry analysis was performed as previously described [11].

### Statistical analysis

The statistical analysis was based on three independent experiments. All samples subjected to real-time PCR were run in triplicate. Statistical analysis was carried out with Student's t-test in Prism 5.0 (GraphPad Software, San Diego, CA, USA). Gray values that were obtained by western blot and Gelatin Zymography were analyzed by ImageJ software. Data were described using mean ± SD. P-values <0.05 (two-sided) were considered statistically significant (ns, not significant, * p< 0.05, ** p<0.01, *** p<0.001).

## SUPPLEMENTARY FIGURES



## References

[1] Han W, Lou Y, Tang J, Zhang Y, Chen Y, Li Y, Gu W, Huang J, Gui L, Tang Y, Li F, Song Q, Di C (2001). Molecular cloning and characterization of chemokine-like factor 1 (CKLF1), a novel human cytokine with unique structure and potential chemotactic activity. BIOCHEM J.

[2] Han W, Ding P, Xu M, Wang L, Rui M, Shi S, Liu Y, Zheng Y, Chen Y, Yang T, Ma D (2003). Identification of eight genes encoding chemokine-like factor superfamily members 1-8 (CKLFSF1-8) by in silico cloning and experimental validation. GENOMICS.

[3] Zhang W, Mendoza MC, Pei X, Ilter D, Mahoney SJ, Zhang Y, Ma D, Blenis J, Wang Y (2012). Down-regulation of CMTM8 induces epithelial-to-mesenchymal transition-like changes via c-MET/extracellular signal-regulated kinase (ERK) signaling. J BIOL CHEM.

[4] Zhang H, Nan X, Li X, Chen Y, Zhang J, Sun L, Han W, Li T (2014). CMTM5 exhibits tumor suppressor activity through promoter methylation in oral squamous cell carcinoma. Biochem Biophys Res Commun.

[5] Li H, Li J, Su Y, Fan Y, Guo X, Li L, Su X, Rong R, Ying J, Mo X, Liu K, Zhang Z, Yang F (2014). A novel 3p22. 3 gene CMTM7 represses oncogenic EGFR signaling and inhibits cancer cell growth. ONCOGENE.

[6] Xie J, Yuan Y, Liu Z, Xiao Y, Zhang X, Qin C, Sheng Z, Xu T, Wang X (2014). CMTM3 is frequently reduced in clear cell renal cell carcinoma and exhibits tumor suppressor activities. CLIN TRANSL ONCOL.

[7] Shao L, Guo X, Plate M, Li T, Wang Y, Ma D, Han W (2009). CMTM5-v1 induces apoptosis in cervical carcinoma cells. Biochem Biophys Res Commun.

[8] Li Z, Xie J, Wu J, Li W, Nie L, Sun X, Tang A, Li X, Liu R, Mei H, Wang F, Wang Z, Gui Y (2014). CMTM3 inhibits human testicular cancer cell growth through inducing cell-cycle arrest and apoptosis. PLOS ONE.

[9] Di Meo S, Airoldi I, Sorrentino C, Zorzoli A, Esposito S, Di Carlo E (2014). Interleukin-30 expression in prostate cancer and its draining lymph nodes correlates with advanced grade and stage. CLIN CANCER RES.

[10] Zhang H, Zhang J, Nan X, Li X, Qu J, Hong Y, Sun L, Chen Y, Li T (2015). CMTM3 inhibits cell growth and migration and predicts favorable survival in oral squamous cell carcinoma. Clin Transl Oncol.

[11] Wang Y, Li J, Cui Y, Li T, Ng KM, Geng H, Li H, Shu XS, Li H, Liu W, Luo B, Zhang Q, Mok TS (2009). CMTM3, located at the critical tumor suppressor locus 16q22. 1, is silenced by CpG methylation in carcinomas and inhibits tumor cell growth through inducing apoptosis. CANCER RES.

[12] Su Y, Lin Y, Zhang L, Liu B, Yuan W, Mo X, Wang X, Li H, Xing X, Cheng X, Dong B, Hu Y, Du H (2014). CMTM3 inhibits cell migration and invasion and correlates with favorable prognosis in gastric cancer. CANCER SCI.

[13] Torre LA, Bray F, Siegel RL, Ferlay J, Lortet-Tieulent J, Jemal A (2015). Global cancer statistics, 2012. CA Cancer J Clin.

[14] Zhang ZY, Ge HY (2013). Micrometastasis in gastric cancer. CANCER LETT.

[15] Zhang Y, Du J, Zheng J, Liu J, Xu R, Shen T, Zhu Y, Chang J, Wang H, Zhang Z, Meng F, Wang Y, Chen Y (2015). EGF-reduced Wnt5a transcription induces epithelial-mesenchymal transition via Arf6-ERK signaling in gastric cancer cells. ONCOTARGET.

[16] Mizuguchi Y, Isse K, Specht S, Lunz JR, Corbitt N, Takizawa T, Demetris AJ (2014). Small proline rich protein 2a in benign and malignant liver disease. HEPATOLOGY.

[17] Lamouille S, Xu J, Derynck R (2014). Molecular mechanisms of epithelial-mesenchymal transition. Nat Rev Mol Cell Biol.

[18] Saitoh M, Endo K, Furuya S, Minami M, Fukasawa A, Imamura T, Miyazawa K (2015). STAT3 integrates cooperative Ras and TGF-beta signals that induce Snail expression. ONCOGENE.

[19] Yang J, Mani SA, Donaher JL, Ramaswamy S, Itzykson RA, Come C, Savagner P, Gitelman I, Richardson A, Weinberg RA (2004). Twist, a master regulator of morphogenesis, plays an essential role in tumor metastasis. CELL.

[20] Ansieau S, Morel AP, Hinkal G, Bastid J, Puisieux A (2010). TWISTing an embryonic transcription factor into an oncoprotein. ONCOGENE.

[21] Li J, Deng Z, Wang Z, Wang D, Zhang L, Su Q, Lai Y, Li B, Luo Z, Chen X, Chen Y, Huang X, Ma J (2015). Zipper-interacting protein kinase promotes epithelial-mesenchymal transition, invasion and metastasis through AKT and NF-kB signaling and is associated with metastasis and poor prognosis in gastric cancer patients. ONCOTARGET.

[22] Yang Y, Yang C, Zhang J (2015). C23 protein meditates bone morphogenetic protein-2-mediated EMT via up-regulation of Erk1/2 and Akt in gastric cancer. MED ONCOL.

[23] Pakala SB, Rayala SK, Wang RA, Ohshiro K, Mudvari P, Reddy SD, Zheng Y, Pires R, Casimiro S, Pillai MR, Costa L, Kumar R (2013). MTA1 promotes STAT3 transcription and pulmonary metastasis in breast cancer. CANCER RES.

[24] Wang Y, Zhou BP (2013). Epithelial-mesenchymal Transition---A Hallmark of Breast Cancer Metastasis. Cancer Hallm.

[25] Zhao D, Besser AH, Wander SA, Sun J, Zhou W, Wang B, Ince T, Durante MA, Guo W, Mills G, Theodorescu D, Slingerland J (2015). Cytoplasmic p27 promotes epithelial-mesenchymal transition and tumor metastasis via STAT3-mediated Twist1 upregulation. ONCOGENE.

[26] Hsu KW, Hsieh RH, Huang KH, Fen-Yau LA, Chi CW, Wang TY, Tseng MJ, Wu KJ, Yeh TS (2012). Activation of the Notch1/STAT3/Twist signaling axis promotes gastric cancer progression. CARCINOGENESIS.

[27] Lee TK, Poon RT, Yuen AP, Ling MT, Kwok WK, Wang XH, Wong YC, Guan XY, Man K, Chau KL, Fan ST (2006). Twist overexpression correlates with hepatocellular carcinoma metastasis through induction of epithelial-mesenchymal transition. CLIN CANCER RES.

[28] Wang L, Lin L, Chen X, Sun L, Liao Y, Huang N, Liao W (2015). Metastasis-associated in colon cancer-1 promotes vasculogenic mimicry in gastric cancer by upregulating TWIST1/2. ONCOTARGET.

[29] Cheng GZ, Zhang WZ, Sun M, Wang Q, Coppola D, Mansour M, Xu LM, Costanzo C, Cheng JQ, Wang LH (2008). Twist is transcriptionally induced by activation of STAT3 and mediates STAT3 oncogenic function. J BIOL CHEM.

[30] Kanai M, Konda Y, Nakajima T, Izumi Y, Kanda N, Nanakin A, Kubohara Y, Chiba T (2003). Differentiation-inducing factor-1 (DIF-1) inhibits STAT3 activity involved in gastric cancer cell proliferation via MEK-ERK-dependent pathway. ONCOGENE.

[31] Lin Z, Zhang C, Zhang M, Xu D, Fang Y, Zhou Z, Chen X, Qin N, Zhang X (2014). Targeting cadherin-17 inactivates Ras/Raf/MEK/ERK signaling and inhibits cell proliferation in gastric cancer. PLOS ONE.

[32] Lim SC, Duong HQ, Parajuli KR, Han SI (2012). Pro-apoptotic role of the MEK/ERK pathway in ursodeoxycholic acid-induced apoptosis in SNU601 gastric cancer cells. ONCOL REP.

[33] Liu WH, Chen MT, Wang ML, Lee YY, Chiou GY, Chien CS, Huang PI, Chen YW, Huang MC, Chiou SH, Shih YH, Ma HI (2015). Cisplatin-selected resistance is associated with increased motility and stem-like properties via activation of STAT3/Snail axis in atypical teratoid/rhabdoid tumor cells. ONCOTARGET.

[34] Tania M, Khan MA, Fu J (2014). Epithelial to mesenchymal transition inducing transcription factors and metastatic cancer. Tumour Biol.

[35] Goswami MT, Reka AK, Kurapati H, Kaza V, Chen J, Standiford TJ, Keshamouni VG (2015). Regulation of complement-dependent cytotoxicity by TGF-beta-induced epithelial-mesenchymal transition. ONCOGENE.

[36] Li L, Han R, Xiao H, Lin C, Wang Y, Liu H, Li K, Chen H, Sun F, Yang Z, Jiang J, He Y (2014). Metformin sensitizes EGFR-TKI-resistant human lung cancer cells in vitro and in vivo through inhibition of IL-6 signaling and EMT reversal. CLIN CANCER RES.

[37] Kim MS, Lee WS, Jeong J, Kim SJ, Jin W (2015). Induction of metastatic potential by TrkB via activation of IL6/JAK2/STAT3 and PI3K/AKT signaling in breast cancer. Oncotarget.

[38] Rodriguez-Barrueco R, Yu J, Saucedo-Cuevas LP, Olivan M, Llobet-Navas D, Putcha P, Castro V, Murga-Penas EM, Collazo-Lorduy A, Castillo-Martin M, Alvarez M, Cordon-Cardo C (2015). Inhibition of the autocrine IL-6-JAK2-STAT3-calprotectin axis as targeted therapy for HR-/HER2+ breast cancers. Genes Dev.

[39] Wu J, Patmore DM, Jousma E, Eaves DW, Breving K, Patel AV, Schwartz EB, Fuchs JR, Cripe TP, Stemmer-Rachamimov AO, Ratner N (2014). EGFR-STAT3 signaling promotes formation of malignant peripheral nerve sheath tumors. ONCOGENE.

[40] Lo HW, Cao X, Zhu H, Ali-Osman F (2008). Constitutively activated STAT3 frequently coexpresses with epidermal growth factor receptor in high-grade gliomas and targeting STAT3 sensitizes them to Iressa and alkylators. CLIN CANCER RES.

[41] Yue P, Zhang X, Paladino D, Sengupta B, Ahmad S, Holloway RW, Ingersoll SB, Turkson J (2012). Hyperactive EGF receptor, Jaks and Stat3 signaling promote enhanced colony-forming ability, motility and migration of cisplatin-resistant ovarian cancer cells. ONCOGENE.

[42] Canadas I, Rojo F, Taus A, Arpi O, Arumi-Uria M, Pijuan L, Menendez S, Zazo S, Domine M, Salido M, Mojal S, Garcia DHA, Rovira A (2014). Targeting epithelial-to-mesenchymal transition with Met inhibitors reverts chemoresistance in small cell lung cancer. CLIN CANCER RES.

[43] Wang R, Li Y, Hou Y, Yang Q, Chen S, Wang X, Wang Z, Yang Y, Chen C, Wang Z, Wu Q (2015). The PDGF-D/miR-106a/Twist1 pathway orchestrates epithelial-mesenchymal transition in gemcitabine resistance hepatoma cells. ONCOTARGET.

[44] Zhang L, Yang J, Cai J, Song X, Deng J, Huang X, Chen D, Yang M, Wery JP, Li S, Wu A, Li Z, Li Z (2013). A subset of gastric cancers with EGFR amplification and overexpression respond to cetuximab therapy. Sci Rep.

[45] Luo BH, Xiong F, Wang JP, Li JH, Zhong M, Liu QL, Luo GQ, Yang XJ, Xiao N, Xie B, Xiao H, Liu RJ, Dong CS (2014). Epidermal growth factor-like domain-containing protein 7 (EGFL7) enhances EGF receptor-AKT signaling, epithelial-mesenchymal transition, and metastasis of gastric cancer cells. PLOS ONE.

[46] Zhang Z, Wang J, Ji D, Wang C, Liu R, Wu Z, Liu L, Zhu D, Chang J, Geng R, Xiong L, Fang Q, Li J (2014). Functional genetic approach identifies MET, HER3, IGF1R, INSR pathways as determinants of lapatinib unresponsiveness in HER2-positive gastric cancer. CLIN CANCER RES.

[47] Nagatsuma AK, Aizawa M, Kuwata T, Doi T, Ohtsu A, Fujii H, Ochiai A (2015). Expression profiles of HER2, EGFR, MET and FGFR2 in a large cohort of patients with gastric adenocarcinoma. GASTRIC CANCER.

[48] Cepero V, Sierra JR, Corso S, Ghiso E, Casorzo L, Perera T, Comoglio PM, Giordano S (2010). MET and KRAS gene amplification mediates acquired resistance to MET tyrosine kinase inhibitors. CANCER RES.

[49] Sanchez-Pulido L, Martin-Belmonte F, Valencia A, Alonso MA (2002). MARVEL: a conserved domain involved in membrane apposition events. TRENDS BIOCHEM SCI.

[50] Kyuno D, Yamaguchi H, Ito T, Kono T, Kimura Y, Imamura M, Konno T, Hirata K, Sawada N, Kojima T (2014). Targeting tight junctions during epithelial to mesenchymal transition in human pancreatic cancer. World J Gastroenterol.

[51] Delic S, Thuy A, Schulze M, Proescholdt MA, Dietrich P, Bosserhoff AK, Riemenschneider MJ (2015). Systematic investigation of CMTM family genes suggests relevance to glioblastoma pathogenesis and CMTM1 and CMTM3 as priority targets. Genes Chromosomes Cancer.

[52] Jenkins NC, Liu T, Cassidy P, Leachman SA, Boucher KM, Goodson AG, Samadashwily G, Grossman D (2011). The p16 (INK4A) tumor suppressor regulates cellular oxidative stress. ONCOGENE.

[53] Suh YA, Post SM, Elizondo-Fraire AC, Maccio DR, Jackson JG, El-Naggar AK, Van Pelt C, Terzian T, Lozano G (2011). Multiple stress signals activate mutant p53 in vivo. CANCER RES.

[54] Levine AJ, Oren M (2009). The first 30 years of p53: growing ever more complex. NAT REV CANCER.

[55] Tam KW, Zhang W, Soh J, Stastny V, Chen M, Sun H, Thu K, Rios JJ, Yang C, Marconett CN, Selamat SA, Laird-Offringa IA, Taguchi A (2013). CDKN2A/p16 inactivation mechanisms and their relationship to smoke exposure and molecular features in non-small-cell lung cancer. J THORAC ONCOL.

