# Supplementary material for: Knockdown of CMTM3 promotes metastasis of gastric cancer via the STAT3/Twist1/EMT signaling pathway
Source: Oncotarget. 2016 Apr 18;7(20):29507–19. doi: 10.18632/oncotarget.8789 (PMC5045413; doi:10.18632/oncotarget.8789)
Supplement: Supplementary file 1 [file oncotarget-07-29507-s001.pdf]

SUPPLEMENTARY FIGURES

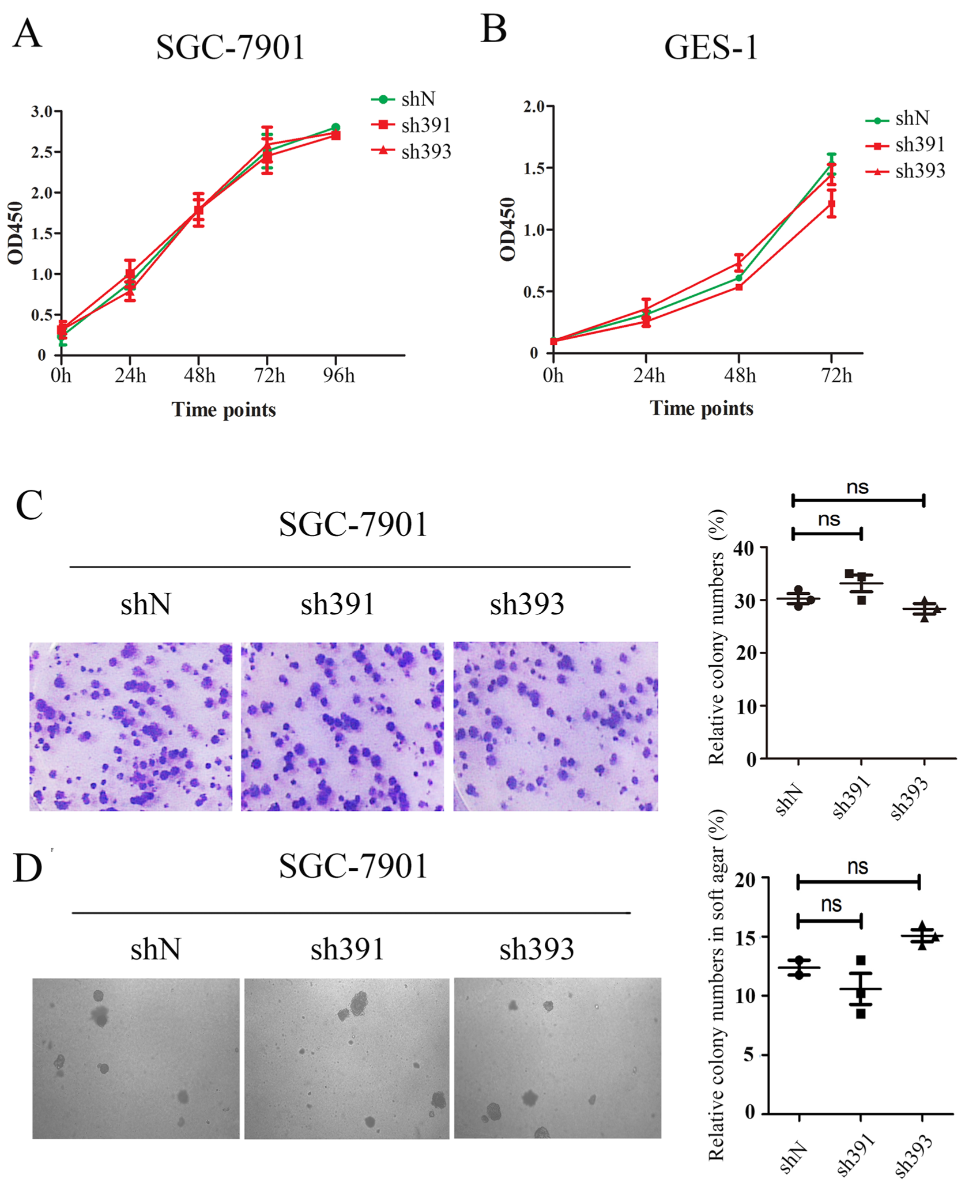

Supplementary Figure S1: Stable knockdown of CMTM3 does not affect cell proliferation. A, B. CCK8 assay of shN, sh391 and sh393 of SGC-7901 and GES-1 cells. **C.** plate colony formation assay of shN and CMTM3-knockdown-SGC-7901 cells; the colonies (≥50) were quantified and representative images of cell colonies were obtained. **D.** soft-agar colony formation assay of shN and CMTM3-knockdown-SGC-7901 cells; the colonies (≥50) were quantified and representative images were obtained (100 × magnification). Data are presented as the mean±s.d (ns, not significant).

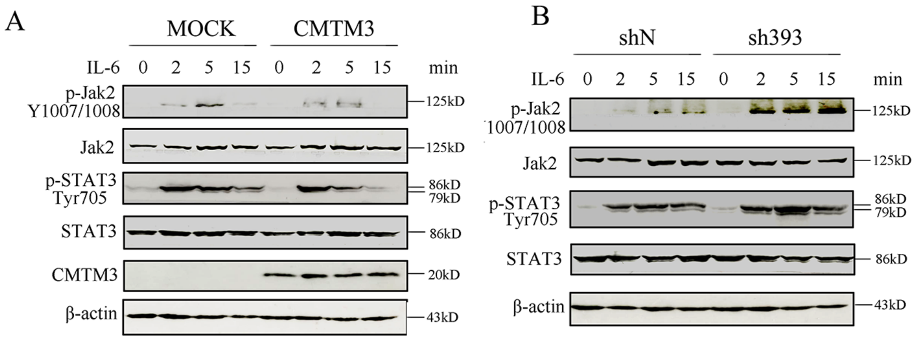

Supplementary Figure S2: CMTM3 has suppressive effects on IL-6 induced Jak2-STAT3 phosphorylation. A. p-Jak2 and p-STAT3 were detected with stimulation of IL-6 (50ng/mL) for different time points in CMTM3 restored SGC-7901 cells. **B.** p-Jak2 and p-STAT3 were detected with stimulation of IL-6 (50ng/mL) for different time points in CMTM3 knockdown SGC-7901 cells.
